# Psychological distress and quality of life in patients with indolent non-Hodgkin lymphoma

**DOI:** 10.1007/s00520-026-10737-4

**Published:** 2026-05-04

**Authors:** Jeanique W. Kuiper, José A. E. Custers, Ankie Kleinjan, Ilse P. G. Verpoorte-Botden, Floortje K. Ploos van Amstel

**Affiliations:** 1 Department of Hematology, Radboud University Medical Center, Nijmegen, The Netherlands; 2 Department of Medical Psychology, Radboud University Medical Center, Nijmegen, The Netherlands; 3 Department of Internal Medicine, Hospital Rivierenland, Tiel, The Netherlands; 4 Department of Medical Oncology, Radboud University Medical Center, Nijmegen, The Netherlands

**Keywords:** Indolent non-Hodgkin lymphoma, HADS, Psychological distress

## Abstract

**Objective:**

Indolent non-Hodgkin lymphoma (iNHL) is a chronic, incurable lymphoid malignancy with a slow course. In absence of symptoms, a ‘watchful waiting’ approach is often chosen. The emotional burden of uncertainty and monitoring may affect patients’ psychological well-being. This study investigates the extent of psychological distress in iNHL patients and its relationship with sociodemographics, clinical characteristics, and quality of life.

**Methods:**

A cross-sectional study was conducted among adults with histologically confirmed indolent B-cell or T-cell NHL, recruited via a regional Dutch hospital and the online patient platform CMyLife. Data were collected through self-administered online questionnaires, including sociodemographics, clinical characteristics, psychological distress (HADS) and quality of life (EORTC QLQ-C30/LG-NHL).

**Results:**

A total of 352 iNHL patients completed the questionnaires (response rate: 37.5%). The mean age was 69 years, 58.5% were male and psychological distress (HADS ≥ 13) was observed in 30.7% of participants. Distressed patients were more often female, less educated, more frequently in the period of watchful waiting, and had more medical comorbidities (p < .01). They reported significantly lower health-related quality of life (HRQoL) (mean 51.2 vs. 77.6) and a higher symptom burden. Psychological distress was strongly correlated with lower HRQoL (r = –.63). In hierarchical regression analysis, psychological distress was the strongest predictor of lower HRQoL (β = –0.605, p < .001).

**Conclusions:**

Approximately one-third of iNHL patients experience psychological distress, significantly associated with female gender, a lower education level, a watchful waiting approach, medical comorbidities, and a lower HRQoL. This underscores the importance of attention for these factors during consultations.

## Introduction

Non-Hodgkin lymphoma (NHL) is a collective term for a heterogeneous group of malignant tumors that originate from lymph nodes and the lymphatic system. NHL is the most common lymphoid malignancy, accounting for 85% of all lymphoid cancers. It is among the ten most common forms of cancer worldwide (Siegel et al. 2018). Approximately 40% of lymphoma diagnoses are indolent diseases, also known as indolent non-Hodgkin lymphoma (iNHL). In the Netherlands, 1,290 people were diagnosed with iNHL in 2022, and 11,414 people who were diagnosed in the past ten years were still alive at that time (Netherlands comprehensive cancer organization (IKNL), z.d.). The incidence of iNHL increases with age, and certain subtypes are associated with viral infections or acquired immune disorders (Kluin-Nelemans & Tansale-Huisman 2013).

iNHL is characterized by a slow and inconspicuous progression. The disease may manifest as a gradual increase in lymphadenopathy, hepatomegaly and/or splenomegaly, as well as cytopenias (Kluin-Nelemans & Tansale-Huisman 2013). In some cases, the detection of the abnormality occurs incidentally during routine medical check-ups or diagnostic procedures for other medical concerns. The diagnosis is typically confirmed through imaging and histological examination of a lymph node biopsy, or via immunophenotyping of peripheral blood. In some cases, bone marrow examination may be necessary.

Treatment is typically initiated when disease-related symptoms occur, such as fatigue, weight loss, or night sweats. In the absence of symptoms, a ‘watchful waiting’ approach is often chosen, carefully monitoring the patient for disease progression, aiming to minimize the impact of treatment side effects and its effect on daily life as long as possible. When treatment is indicated, iNHL generally responds well to therapy, but it has the tendency to relapse. Treatment after relapse is possible and often successful, but these types of lymphoid malignancies are not curable yet (Bachy & Salles 2014; Smyth et al. 2023).

Due to advancements in the treatment of iNHL, particularly with the introduction of the monoclonal antibody rituximab, treatment outcomes have improved drastically, leading to an increase in survival rates. The relative five-year survival rate for patients with iNHL has risen from 58% in 2002 to 86% in 2022 (IKNL, z.d.). In recent years, advances such as bispecific antibodies and Chimeric Antigen Receptor (CAR) T-cell therapy have contributed to the development of therapeutic strategies with the potential to further enhance disease control in the future. Additionally, prognostic indexes are used to better predict survival and tailoring of treatments, taking into account functional status and comorbidities. These advancements have resulted in a substantial group of patients living with iNHL for extended periods, with an expected increase in this number due to the aging global population and improved treatment options (IKNL, z.d.; Ekels et al. 2022).

Due to the indolent character of the disease and the increasing survival rates, iNHL becomes a chronic illness for many patients. Patients cannot be cured but also do not die directly from the disease. However, in this prolonged lifespan patients may experience psychological difficulties. Receiving a cancer diagnosis, its treatment and consequences are considered to be stressful experiences, which can affect the psychological well-being of patients. According to the stress-coping theory by Lazarus and Folkman (1984), psychological well-being is determined as the balance between stress factors and available resources to cope with them. Resources can include emotional support from loved ones, professional help from healthcare providers, and the personal resilience of the patient. Some patients adapt well to the disease and experience little psychological issues. However, a significant percentage of patients continues to struggle with persistent feelings of anxiety and depression, also known as psychological distress (Oerlemans et al. 2014; Raphael et al., 2017).

Psychological distress can lead to a lower health-related quality of life (HRQoL) and increased healthcare use (Oerlemans et al. 2014; Arts et al. 2018). Patients with iNHL may experience psychological distress due to the chronic character of their disease, the prospect of disease progression, and repeated treatments. The uncertainty about the future of the disease and the periods of watchful waiting can amplify feelings of tension and uncertainty. This watchful waiting approach, which contrasts with the more aggressive treatment strategies used for other types of cancer, may lead patients to feel that insufficient efforts are being made to treat the disease. The experience of uncertainty can be exacerbated by the feeling that healthcare providers are underestimating the severity of the situation (Sheridan et al. 2023).

Extensive research has been conducted on the prevalence and contributing factors of psychological distress in patients with certain types of cancer, particularly in solid oncology (Duarte et al. 2022; Lopes et al. 2022; Yeung et al. 2024). However, there is limited knowledge regarding psychological distress in patients with iNHL. Given the nature of this disease, which is chronic and often managed through periods of watchful waiting, it is plausible that similar patterns of psychological distress are present in this hematological group as well. Gaining insight into its prevalence and predisposing factors can help healthcare providers prevent or manage psychological distress in this patient group. This study aims to investigate the extent of psychological distress in iNHL patients and its relationship with sociodemographics, clinical characteristics, and quality of life.

## Methods

### Study design

A cross-sectional study was conducted between January 2024 and April 2024.

### Participants

Patients with a histologically confirmed indolent B-cell or T-cell lymphoma, as defined by the International Classification of Diseases for Oncology-3 codes (ICD 0—3) were eligible to participate. Patients were recruited 1) through a regional hospital in the Netherlands or 2) via the online patient platform CMyLife. CMyLife is a patient platform established by hematologists, patients, and patient organizations with the goal of improving the quality of care for chronic hematological patients. Patients had to be 18 years or older. Patients with serious cognitive impairment (e.g., dementia) were excluded.

### Recruitment procedure

Potential participants from the regional hospital were selected through a search in the electronic patient file, based on diagnosis-treatment combination codes for iNHL. The resulting patient list was sorted by patient number and manually screened to verify whether patients were still receiving care at the regional hospital and had not passed away. Eligible patients received an email invitation including study information and informed consent. After informed consent, patients completed the online questionnaires. For patients using the CMyLife patient platform, a link to the digital questionnaires was shared through news articles on the website and newsletters.

Ethical approval for this study was granted by the Ethical Committee Research (ECO) of HAN University of Applied Sciences. As the study did not fall under the Medical Research Involving Human Subjects Act (WMO), informed consent was obtained through the initial question in the questionnaire, in accordance with the General Data Protection Regulation (GDPR). As a member of the treatment team, the researcher had access to patient files and email addresses of the patients treated at the regional hospital. Patients who create an account on the CMyLife patient platform consent to being approached for scientific research. A patient information letter was included in the data collection platform. The study was conducted in accordance with the Dutch Central Committee on Research Involving Human Subjects (CCMO) guidelines for research not subject to the WMO, and with the Declaration of Helsinki.

### Measures

Sociodemographic and clinical characteristics for all participants in this study were obtained through self-report using questionnaires. The questionnaires collected information on age, gender, marital status, education level, and employment status. Self-reported clinical characteristics included type of diagnosis, time since diagnosis, treatment status, and medical comorbidities. Medical comorbidities were identified using the Self-administered Comorbidity Questionnaire (SCQ) (Sangha et al., 2003). Participants were asked to report comorbid conditions present in the past twelve months: stroke, hypertension, pulmonary disease, diabetes, stomach or intestinal diseases, kidney disease, liver disease, thyroid problems, rheumatic conditions, and cardiac disease. The responses were summed, and the total score was categorized into three groups: no comorbidity, 1 comorbidity, or ≥ 2 comorbidities.

*Psychological distress* was assessed using the Hospital Anxiety and Depression Scale (HADS) (Zigmond & Snaith 1983). Each item is rated on a four-point scale from 0 to 3. The total score was calculated by summing the item scores, ranging from 0 to 42, with higher scores indicating more psychological distress. A cut-off score for psychological distress of HADS total score of ≥ 13 was used (Arts et al. 2021).

*Quality of life* was assessed using the Dutch version of the European Organization for Research and Treatment of Cancer Quality of Life Questionnaire-Core30 (EORTC QLQ-C30) (Aaronson et al. 1993). This questionnaire includes functional scales, symptom scales, and two questions regarding health status and quality of life. Scores were calculated and transformed on a scale from 0 to 100. Higher scores on the functional and general scales indicate an improved level of functioning and better health-related quality of life, whereas higher scores on the symptom scales reflect a higher burden of symptoms. In addition, a summary score was calculated from the mean of the functioning and symptom scales, except for global QoL and the financial impact scale. A high score on the summary score represents a better quality of life (Husson et al. 2020).

For *disease-specific aspects of quality of life*, the Dutch version of the EORTC QLQ Low Grade Non-Hodgkin Lymphoma (LG-NHL) questionnaire was used. This questionnaire, specifically developed for patients with indolent non-Hodgkin lymphomas, measures symptom burden, physical condition, emotional impact, and concerns related to health and functioning (Oerlemans et al. 2023). Scores were similarly calculated as for the EORTC QLQ-C30, with higher scores indicating a higher degree of symptomatology or problems.

### Statistical analyses

Analyses were performed using the IBM Statistical package for the Social Science (SPSS) version 28 (IBM Corp.(2021) Armonk, NY, USA). A p-value of < 0.05 was considered statistically significant. Patients with incomplete questionnaire responses were excluded from the analysis. Differences in sociodemographics and clinical characteristics between patients from the regional hospital and CMyLife platform, as well as non-distressed and distressed iNHL patients, were compared using Chi-square tests for nominal variables and Mann–Whitney tests for ordinal variables. A one-way ANOVA was conducted to identify sociodemographics and clinical characteristics associated with psychological distress. A multivariate analysis of variance (MANOVA) was performed to examine overall differences in quality of life between non-distressed and distressed iNHL patients across the domains of the EORTC QLQ-C30 and the QLQ-LG20. Psychological distress (non-distressed versus distressed) was included as the independent variable, and the aspects of quality of life were included as dependent variables. Mean HRQoL scores were compared between non-distressed and distressed iNHL patients. A difference greater than 10 points was considered clinically relevant, based on the criteria established by Osoba et al. (1998), who described changes of 5—10 points as small, 10—20 points as moderate, and over 20 points as large. The Pearson correlation coefficient was calculated to determine the strength of the association between psychological distress and quality of life. A correlation coefficient (r) of 0.10-0.29 was considered weak, 0.30-0.49 moderate, and ≥ 0.50 strong (Cohen 1988). Additionally, a hierarchical regression analysis was performed with the EORTC QLQ-C30 summary score as the dependent variable. In this analysis, sociodemographics (gender, age, education level) were entered first, medical variables (time since diagnosis, treatment status, medical comorbidity) second, and psychological distress (HADS total) third.

## Results

### Patients’ characteristics

For this study, 940 iNHL patients were contacted, including 180 patients under treatment at the regional hospital and 760 patients from other parts of the Netherlands using the CMyLife patient platform. Of these, 352 patients completed the questionnaire (37.5%). The sociodemographics and clinical characteristics of the patients are summarized in Table 1. Mean age of the total study sample was 69 years (SD 8.8). Most of the patients were male (58.5%), married or living with a partner (83.5%), and retired (63.6%). The most common diagnosis was chronic lymphocytic leukemia/small lymphocytic lymphoma (80.7%), with 44.0% of patients having a time since diagnosis of 2—5 years. Most of the patients were during the period of watchful waiting (76.4%). Approximately half of the patients reported at least one medical comorbidity, with hypertension (21.3%), pulmonary disease (20.0%), and cardiac disease (17.0%) being the most prevalent.

**Table 1:** Sociodemographics and clinical characteristics of the total study population

	Total *N* = 352	Regional *n* = 127	CMyLife *n* = 225	*p* value
**Gender**, n (%)				
Male	206 (58.5%)	71 (55.9%) 56 (44.1%)	135 (60.0%)	.454^1^
Female	146 (41.5%)		90 (40.0%)	
**Age**, mean (SD) in years	69 (8.8)	71 (9.1)	68 (8.4)	** < .001**^**2**^
**Marital status**, n (%)				
Relationship	22 (6.3%)	108 (85.0%)	186 (82.7%)	.535^3^
Divorced	29 (8.2%)	5 (3.9%)	17 (7.6%)	
Widowed	7 (2.0%)	12 (9.4%)	17 (7.6%)	
Single	294 (83.5%)	2 (1.6%)	5 (2.2%)	
Education level*, n (%)				
Low	43 (12.3%)	29 (22.9%)	14 (6.2%)	** < .001**^**4**^
Medium	114 (32.3%)	48 (37.8%)	66 (29.3%)	
High	195 (55.4%)	50 (39.3%)	145 (64.5%)	
**Employment**, n (%)				
Paid work	90 (25.0%)	26 (20.5%)	64 (28.4%)	.259^3^
Unemployed	17 (4.8%)	5 (3.9%)	12 (5.3%)	
Unpaid work/voluntary work	7 (2.0%)	3 (2.4%)	4 (1.7%)	
Disability insurance	14 (4.0%)	3 (2.4%)	11 (4.9%)	
Retirement	224 (63.6%)	90 (70.9%)	134 (59.6%)	
**Cancer type**, n (%)				
Follicular lymphoma	20 (5.7%)	19 (15.0%)	1 (0.4%)	< .001^3#^
CLL/SLL	284 (80.7%)	66 (52.0%)	218 (96.9%)	
Hairy-cell-leukemia	3 (0.9%)	3 (2.4%)	0 (0.0%)	
Marginal zone lymphoma	14 (4.0%)	14 (11.0%)	0 (0.0%)	
Waldenström’s macroglobulinemia	14 (4.0%)	11 (8.7%)	3 (1.3%)	
Otherwise/I don’t know	17 (4.8%)	14 (11.0%)	3 (1.3%)	
**Time elapsed since diagnosis**, n (%)				
< 2 years	59 (16.8%)	32 (25.2%)	27 (12.0%)	** < .001**^**4**^
2—5 years	155 (44.0%)	60 (47.2%)	95 (42.2%)	
> 5 years	138 (39.2%)	35 (27.6%)	103 (45.8%)	
**Treatment**, n (%)				
Active treatment	83 (23.6%)	23 (18.1%)	60 (26.7%)	.069^1^
Watchful waiting	269 (76.4%)	104 (81.9%)	165 (73.3%)	
**Medical comorbidities**, n (%)				
No comorbidities	50 (21.3%)	17 (32.7%)	33 (31.4%)	.878^1^
1 comorbidity	47 (20.0%)	14 (26.9%)	33 (31.4%)	
≥ 2 comorbidities	40 (17.0%)	14 (26.9%)	26 (24.8%)	
**Most frequent comorbidities**, n (%)				
Hypertension	195 (55.4%)	75 (59.1%)	120 (53.5%)	.741
Pulmonary disease	104 (29.5%)	34 (26.8%)	70 (31.1%)	.335
Cardiac disease	53 (15.1%)	8 (14.1%)	35 (15.5%)	.880

Abbreviations: CLL/SLL = chronic lymphocytic leukemia/small lymphocytic lymphoma

^*^Education levels: low = primary school, lower vocational; medium = secondary school, secondary vocational; high = higher general, higher vocational, university

^1^ Chi-Square test. ^2^ Independent Samples T-test. ^3^ Fisher’s exact test. ^4^ Mann–Whitney test

Bold numbers indicate statistical significance (p < .05). ^#^Low cell count; p-value not interpreted as significant

In total, 30.7% of the patients scored above the cut-off (≥ 13) for psychological distress (*n* = 108) (Table 2). The prevalence of psychological distress was higher among patients from the regional hospital compared to the CMyLife patient population (41.7% versus 24.4%; p < 0.001).

**Table 2:** Incidence of psychological distress of the total study population, including regional and national

	Total *N* = 352	Regional *n* = 127	CMyLife *n* = 225	p value
**Psychological distress**, n (%)				** < .001**^1^
Non-distressed	244 (69.3%)	74 (58.3%)	170 (75.6%)	
Distressed	108 (30.7%)	53 (41.7%)	55 (24.4%)	

^1^ Chi-Square test. Bold numbers indicate statistical significance (p < .05)

### Associations between non-distressed and distressed iNHL patients and sociodemographics and clinical characteristics

Patients who scored above the cutoff for psychological distress were more often female (54.6% versus 45.4%; p < 0.001), had less frequently attained a higher education (39.8% versus 62.3%; p = 0.002), were more often during the period of watchful waiting (85.9% versus 66.6%; p < 0.001), and reported more medical comorbidities (Table 3).

**Table 3:** Associations between non-distressed (HADS-T < 13) and distressed (HADS-T ≥ 13) lymphoma patients and sociodemographics and clinical characteristics

	Non-distressed n = 244	Distressed n = 108	p value
**Sociodemographics**			
**Gender, n (%)**			
Male Female	157 (64.3%) 87 (35.7%)	49 (45.4%) 59 (54.6%)	** < .001**^**1**^
**Age**, mean (SD) in years	69 (8.6)	68 (9.0)	.072^2^
**Marital status**, n (%)			
Relationship Divorced Widowed Single	207 (84.8%) 10 (4.1%) 23 (9.4%) 4 (1.6%)	87 (80.6%) 12 (11.1%) 6 (5.6%) 3 (2.8%)	.058^3^
**Education level**, n (%)			
Low Medium High	25 (10.2%) 67 (27.4%) 152 (62.3%)	18 (16.6%) 47 (43.5%) 43 (39.8%)	**.002**^**4**^
**Clinical characteristics**			
**Time elapsed since diagnosis**, n (%)			
< 2 years 2—5 years > 5 years	33 (13.5%) 113 (46.3%) 98 (40.2%)	26 (24.1%) 42 (38.9%) 40 (37.0%)	.057^4^
**Treatment**, n (%)			
Active treatment Watchful waiting	67 (33.4%) 177 (66.6%)	16 (14.1%) 92 (85.9%)	** < .001**^**1**^
**Comorbidities**, n (%)			
No comorbidities 1 comorbidity ≥ 2 comorbidities	146 (59.8%) 73 (29.9%) 25 (10.3%)	49 (45.4%) 31 (28.7%) 28 (25.9%)	**.003**^**1**^

Abbreviations: HADS-T = Hospital Anxiety and Depression scale total score

^*^Education levels: low = primary school, lower vocational; medium = secondary school, secondary vocational; high = higher general, higher vocational, university

^1^ Chi-Square test. ^2^ Independent Samples T-test. ^3^ Fisher’s exact test. ^4^ Mann–Whitney test

Bold numbers indicate statistical significance (p < .05)

No statistically significant differences were found regarding age, marital status, or time since diagnosis (p > 0.05).

### Associations between non-distressed and distressed iNHL patients and quality of life

Multivariate analyses of variance (MANOVAs) were conducted to examine overall differences in quality of life between non-distressed and distressed iNHL patients. The MANOVA for the EORTC QLQ-C30 showed a significant overall effect of psychological distress on quality of life, Wilks’ Λ = 0.506, F(14, 337) = 23.53, p < 0.001, partial η^2^ = 0.49. Similarly, the MANOVA for the EORTC QLQ-LG20 showed a significant overall effect, Wilks’ Λ = 0.639, F(4, 347) = 49.09, p < 0.001, partial η^2^ = 0.36. The between-subjects effects are presented in Table 4. iNHL patients with high levels of psychological distress reported a lower mean health-related quality of life compared to those with low levels of psychological distress (51.2 versus 77.6). A strong correlation (r = -0.63) was observed between health-related quality of life and psychological distress. The differences between non-distressed and distressed iNHL patients were significant across all functional scales of the QLQ-C30 and were significantly correlated with psychological distress (p < 0.001). Psychological distress was associated with lower subscale scores, indicating reduced functional capacities. The subscale scores of the symptom scales, as well as the disease-specific questions (QLQ-LG), showed significant differences between non-distressed and distressed iNHL patients. These scores also displayed significant correlations with psychological distress (p < 0.001). Psychological distress was associated with higher subscale scores, indicating a higher symptom burden.

**Table 4:** Associations between non-distressed (HADS-T < 13) and distressed (HADS-T ≥ 13) lymphoma patients and quality of life

	Non-distressed *n* = 244	Distressed *n* = 108	*p* value (between-subjects effects)	Δ Score	Pearson’s correlation
**EORTC QLQ-C30**, mean (SD)					
**Function scales**					
Physical functioning Role functioning Emotional functioning Cognitive functioning Social functioning	87.6 (15.2) 84.2 (21.1) 88.1 (12.5) 84.2 (17.1) 88.0 (20.0)	72.8 (18.4) 67.3 (25.0) 61.7 (15.6) 69.6 (22.9) 65.4 (26.3)	** < .001** ** < .001** ** < .001** ** < .001** ** < .001**	14.8* 16.9* 26.4* 14.6* 22.6*	-.499** -.438** -.798** -.414** -.473**
**Symptom scales**					
Fatigue Nausea/vomiting Pain Dyspnea Insomnia Appetite loss Constipation Diarrhea Financial difficulties	24.0 (19.7) 3.1 (9.8) 8.4 (15.9) 14.8 (21.8) 19.9 (22.9) 5.7 (15.2) 7.1 (16.1) 7.0 (16.6) 5.2 (16.8)	46.0 (22.3) 9.0 (14.1) 25.6 (26.6) 26.9 (27.5) 42.9 (31.9) 17.0 (23.9) 16.4 (26.0) 14.5 (24.7) 9.0 (22.6)	** < .001** ** < .001** ** < .001** ** < .001** ** < .001** ** < .001** ** < .001** **.004** .123	22.0* 5.9 17.2* 12.1* 23.0* 11.3* 9.1 7.5 3.8	.573** .239** .392** .308** .415** .305** .242** .199** .134
Health related quality of life	77.6 (18.6)	51.2 (19.8)	** < .001**	26.4*	-.634**
Summary score	87.9 (9.9)	72.2 (12.7)	** < .001**	15.7*	
**EORTC QLQ-LG20,** mean (SD)					
Symptom burden Physical condition/fatigue Emotional impact Worries health/functioning Problems with work/study Fears about work/study	13.5 (13.0) 20.5 (18.1) 14.5 (15.6) 23.9 (16.0) 10.3 (22.8) 9.5 (20.9)	26.9 (19.0) 40.4 (21.3) 40.0 (21.3) 44.8 (20.6) 22.0 (33.6) 25.1 (36.0)	** < .001** ** < .001** ** < .001** ** < .001** ** < .001** .005	13.4* 19.9* 25.5* 20.9* 11.7* 15.6*	.446** .546** .692** .782** .240** .295**

Abbreviations: HADS-T = Hospital Anxiety and Depression scale total score, EORTC QLQ-C30 = European Organization for Research and Treatment of Cancer Quality of Life Questionnaire C30, QLQ-LG20 = Quality of Life Questionnaire Low Grade Non-Hodgkin Lymphoma

P-values show which specific dependent variables are significantly different between non-distressed and distressed iNHL patients (MANOVA). Pearson’s correlation coefficients (r) represent the strength of the association between HADS total scores and quality of life scores

Bold numbers indicate statistical significance (p < .05)

^*^Clinically relevant difference: ≥ 10 points (Osoba et al. 1998)

^**^Correlation is significant on the .01 level

No statistically significant differences were found between the two groups on the financial difficulties subscale (p > 0.05).

A hierarchical multiple regression was conducted to examine the association of medical factors and psychological distress with quality of life, after controlling for sociodemographics (age, gender and education level). The results are presented in Table 5. Age, gender and education level were entered at step 1, explaining 5.3% of the variance in quality of life (EORTC QLQ-C30 summary score). After the entry of medical variables (time since diagnosis, treatment status and medical comorbidity) at step 2, the total variance explained by the model increased to 16.7%, F(6, 345) = 11.554, p < 0.001. The medical variables explained an additional 11.5% of the variance in quality of life, after controlling for sociodemographic factors (R^2^ change = 0.115, F change (3, 345) = 11.554, p < 0.001). At step 3, psychological distress (HADS total) was entered into the model, and the total variance explained by the model as a whole was 48.6%, F(7, 344) = 46.513, p < 0.001. The inclusion of psychological distress explained an additional 31.9% of the variance in quality of life, after accounting for sociodemographic and medical factors (R^2^ change = 0.319, F change (1, 344) = 46.513, p < 0.001). In the final model medical comorbidity, treatment status and psychological distress were statistically significant. Psychological distress showed the strongest association, with a beta (β) value of -0.605 (p < 0.005), followed by medical comorbidity (β = -0.201, p < 0.001) and treatment status – coded as watchful waiting versus active treatment – (β = 0.095, p = 0.015), indicating that patients during the period of watchful waiting reported lower quality of life than those receiving active treatment.

**Table 5:** Hierarchical regression analysis on associated variables of quality of life (EORTC QLQ-C30 summary score)

Predictor	Model 1 β	Model 2 β	Model 3 β
Age (years)	-0.009	0.023	-0.019
Gender	-0.203***	-0.151**	-0.035
Education level	0.106*	0.116*	0.003
Treatment	–	0.144**	0.095*
Time since diagnosis	–	0.009	-0.019
Comorbidities	–	-0.308***	-0.201***
HADS total	–	–	-0.605***
**Model fit**			
	**Model 1**	**Model 2**	**Model 3**
R^2^	**.053**	.167	.486
ΔR^2^	–	.115***	.319***
F	6.44***	11.55***	46.51***

Abbreviations: HADS-T = Hospital Anxiety and Depression scale total score, EORTC QLQ-C30 = European Organization for Research and Treatment of Cancer Quality of Life Questionnaire C30

β = standardized regression coefficients

^*^p < .05

^**^p < .01

^***^p < .001

Age, gender, education level and time since diagnosis did not significantly contribute to the final model (p > 0.05).

## Discussion

This cross-sectional study showed that one-third (30.7%) of iNHL patients experience elevated levels of psychological distress, with a higher prevalence in the regional sample. Additionally, psychological distress was associated with female gender, a lower education level, watchful waiting approach, medical comorbidity, and quality of life.

The reported prevalence of psychological distress in this study is higher than that found in previous research among patients with lymphoma. Tilch et al. (2022) reported a prevalence of 24.0% among patients with Hodgkin lymphoma (HL) and diffuse large B-cell lymphoma (DLBCL), while Arts et al. (2021) found a prevalence of 17.0% in patients with Hodgkin lymphoma (HL), non-Hodgkin lymphoma (NHL), and chronic lymphocytic leukemia (CLL). In general, studies conducted in oncology populations report a prevalence of psychological distress of approximately one in three patients (Zabora et al. 2001; Mitchell et al. 2011), which is consistent with the findings of the present study. Variations in tumor types and the cutoff scores used to define psychological distress may contribute to differences in reported prevalence across studies.

When looking at sociodemographics and clinical characteristics, the results of this study show that females, patients with lower education levels, patients during the period of watchful waiting, and those with medical comorbidities experienced more psychological distress. Previous studies have indicated that women are at higher risk for psychological distress (Götze et al. 2020; Agostinelli et al. 2022; Carter et al. 2025)). Patients with lower education levels may experience more socio-economic stressors, which could also contribute to psychological distress (Tilch et al. 2022; Tang et al. 2024). In this study, the patients of CMyLife had on average, a higher education level compared to the patients of the regional hospital, which may have contributed to the lower prevalence of psychological distress in this group. Patients during the period of watchful waiting also experienced more psychological distress. The uncertainty associated with this treatment modality may contribute to feelings of tension and anxiety and can be emotionally burdening for the patient (Howell et al. 2022). Additionally, patients without active treatment may receive less social support due to less visibility of their disease, which can increase their psychological burden (Watts et al. 2014; Trevino et al. 2022). A study by Leukaemia Care (2019) involving 1,152 patients with hematological cancers, found that those in the period of watchful waiting were significantly less likely to receive information about emotional support (39% versus 69%) or to have access to a clinical nurse specialist, despite these being identified as key drivers for an improved patient experience (Leukaemia Care 2019). These findings highlight potential challenges for patients in the period of watchful waiting, who typically have less frequent contact with healthcare providers compared to those undergoing active treatment. Reduced access to psychological support during this period may contribute to increased levels of psychological distress. Medical comorbidity was also associated with psychological distress. The presence of comorbidities may interact with cancer, resulting in a greater overall symptom and emotional burden (Petrova et al. 2021). No association was found between age and psychological distress. The lack of correlation may be due to the relatively small variation in the ages of respondents, as most participants were older patients. While literature suggests that older individuals might have better coping mechanisms due to more life experience (Kuba et al. 2019), the limited age range in this study may have restricted the ability to detect any potential relationship between age and psychological distress. Additionally, the number of years since diagnosis was not significantly related to psychological distress, suggesting that distress is not confined to the initial years of following diagnosis. However, findings on the timing of psychological distress among cancer patients and survivors have been mixed. While some studies found no significant differences in psychological distress between short-term and long-term survivors (Troy et al. 2019; Götze et al. 2020), others indicated that psychological distress is higher among patients with a shorter time since diagnosis (Tang et al. 2024; Odejide et al. 2025). For patients with chronic cancers like iNHL, the experience may differ from that of survivors, as they continue to live with the disease over an extended period. The uncertainty about disease progression, treatments, and fluctuating symptom burden may lead to a persistent or evolving psychological strain resulting in long-term emotional exhaustion and heightened vulnerability to psychological distress.

As expected, psychological distress was strongly associated with a lower quality of life. Distressed patients scored lower on health-related quality of life, functional scales, and expressed more concerns about their health and functioning. This confirms findings from previous studies (Oerlemans et al. 2014; Tilch et al. 2022). Psychological distress was accompanied by a higher symptom burden, suggesting that distress and physical symptoms may influence each other (Holtzer-Goor et al. 2015; Youron et al. 2020). However, while distress could potentially exacerbate physical symptoms, it is also possible that physical discomfort contributes to greater psychological distress. In this study, a large clinically relevant difference in health-related quality of life was found between non-distressed and distressed lymphoma patients.

Additionally, the hierarchical regression analysis further demonstrated the significant relationship between psychological distress and quality of life. After accounting for sociodemographics and medical factors, psychological distress was the strongest predictor in the final model, explaining 31.9% of the variance in quality of life. This finding is in line with previous studies that also identified psychological distress as a key determinant of quality of life among cancer patients (Yao et al. 2024; Caldiroli et al. 2025).

Both non-distressed and distressed iNHL patients reported fatigue and concerns about health and functioning as the most common issues. According to research by the Dutch Federation of Cancer Patient Organizations (NFK), a significant majority (85%) of people with cancer (or who have had cancer) experience worry at some point because of their disease. These feelings have a substantial impact on the quality of life of 70% of these individuals (NFK, 2023). Furthermore, fatigue is one of the most prevalent problems among cancer patients, often associated with treatment (Ekels et al. 2022). However, despite iNHL generally being considered asymptomatic during the period of watchful waiting, the findings of this study suggest that patients may still experience physical symptoms, such as fatigue. This challenges the assumption that the period of watchful waiting is completely symptom-free.

### Study limitations

This study had several limitations. The cross-sectional design does not allow claims of causality or does it provide insight into the course of psychological distress over time. Additionally, no distinction was made between the different subtypes of iNHL, which could be a confounder since various subtypes may have different outcomes. Furthermore, the questionnaires could only be completed online, which may have introduced a bias towards digitally skilled participants. Moreover, the relatively low response rate (37.5%) may have led to participation bias, as participants may differ from non-participants in relevant characteristics such as health status or psychological distress. As no data were available for non-participants, we were unable to formally assess potential differences, which may limit the generalizability of the findings. A further limitation of this study is the lack of a theoretically driven conceptual model guiding the analyses. Consequently, the relationships between potentially modifiable factors and psychological distress were examined in an exploratory manner, which leaves open the possibility that other important variables are involved and findings should be confirmed in future research.

A key strength of this study is its large and diverse patient population, encompassing both regional and national settings, enabling broad generalizability of the findings for iNHL patients.

### Clinical implications

Given the association with modifiable risk factors, it is important to identify psychological distress at an early stage. The systematic use of standardized screening tools during consultations – such as questionnaires focusing on anxiety, depression, or general distress – can help healthcare professionals identify vulnerable patients, enabling timely and targeted psychosocial support. Furthermore, the strong relationship between psychological distress and lower quality of life suggests that interventions effectively aimed at reducing distress could have a significant positive impact on the daily functioning and well-being of patients with iNHL. To further inform clinical practice, future studies are necessary to explore the prevalence of psychological distress in different subtypes of iNHL. Longitudinal studies are essential to gain a more accurate understanding of the development of psychological distress over time in iNHL patients.

## Conclusion

In conclusion, approximately one-third of iNHL patients experience psychological distress. This psychological distress is significantly associated with female gender, a lower education level, a watchful waiting approach, medical comorbid conditions, and a lower health-related quality of life. This underscores the importance of attention for these factors during consultations.

## Data Availability

No datasets were generated or analysed during the current study.
